# Unilateral hilar sarcoidosis with anemia and low T3 syndrome: a case report

**DOI:** 10.3389/fmed.2026.1892525

**Published:** 2026-07-08

**Authors:** Yuexiang Shui, Huabin Wang, Shaobin Wang

**Affiliations:** 1 Department of Respiratory and Critical Care Medicine, Lanxi People’s Hospital, Lanxi, Zhejiang, China; 2 Department of Clinical Laboratory, Affiliated Jinhua Hospital, Zhejiang University School of Medicine, Jinhua, Zhejiang, China; 3 The Second Affiliated Hospital Zhejiang University School of Medicine, Zhejiang Respiratory Diseases Research Institute, Hangzhou, Zhejiang, China

**Keywords:** endobronchial ultrasound-guided transbronchial needle aspiration, iron metabolism, low T3 syndrome, sarcoidosis, unilateral hilar lymphadenopathy

## Abstract

**Background:**

Sarcoidosis is a systemic granulomatous disease that typically presents with bilateral hilar or mediastinal lymphadenopathy. Unilateral hilar involvement is uncommon and may closely mimic lung cancer, lymphoma, or tuberculosis. Inflammatory activation in sarcoidosis may also disrupt iron metabolism and thyroid hormone homeostasis, but these extra-radiological clues are often overlooked in routine practice.

**Case presentation:**

A 53-year-old woman was admitted with fever, chest tightness, and a left hilar mass with mediastinal lymphadenopathy. Laboratory tests showed pancytopenia and markedly elevated inflammatory markers, including interleukin-6 at 60.7 pg/mL and C-reactive protein at 98.7 mg/L. Iron studies showed a discordant profile: low serum iron of 6.3 μmol/L, reduced transferrin, and markedly elevated ferritin of 955.2 μg/L. Total T3 was also low at 0.57 nmol/L. She had a 2-year history of anemia with poor response to oral iron therapy. Initial endobronchial ultrasound-guided transbronchial needle aspiration (EBUS-TBNA) of station seven lymph nodes was non-diagnostic. Because malignancy and infection remained possible, repeat EBUS-TBNA targeting stations 4L and 11L was performed and showed non-caseating granulomatous inflammation. Microbiological tests for tuberculosis and fungal infection were negative, and tumor markers did not support malignancy. Intrathoracic sarcoidosis with atypical unilateral hilar and mediastinal lymphadenopathy was diagnosed. The anemia was interpreted as mixed absolute iron deficiency and inflammation-related iron restriction, accompanied by low T3 syndrome in the setting of systemic inflammation. Treatment with intravenous iron sucrose and inhaled budesonide-formoterol was followed by regression of the hilar and mediastinal lesions, normalization of hemoglobin, and recovery of T3 levels.

**Conclusion:**

The case highlights the diagnostic challenge of unilateral hilar sarcoidosis, which may mimic malignancy or infection. When initial EBUS-TBNA is non-diagnostic but clinical suspicion persists, repeat multi-station sampling should be considered in selected cases to reduce false-negative interpretation. The accompanying anemia and low T3 syndrome may provide supportive evidence of systemic inflammation, but they should not be regarded as central diagnostic features. Continued follow-up is warranted to monitor the clinical course.

## 1 Introduction

Sarcoidosis is a systemic granulomatous disorder of uncertain etiology, and current evidence suggests that its development likely results from the interaction between genetic susceptibility and environmental antigen exposure (1). Histopathologically, it is characterized by non-caseating granulomas and can involve multiple organs, most commonly the lungs and intrathoracic lymph nodes, but also the skin, heart, and other extrapulmonary sites (2). Thoracic involvement is common, but atypical unilateral hilar or mediastinal disease may create substantial diagnostic uncertainty at initial presentation (3). Radiologically, thoracic sarcoidosis typically presents with bilateral hilar and mediastinal lymphadenopathy, often with a symmetric distribution (3). Supportive CT features include well-defined lymph nodes, smooth margins, preservation of adjacent bronchovascular structures, and absence of necrosis or cystic change (4, 5). However, unilateral hilar or mediastinal lymphadenopathy is uncommon and may mimic lung cancer, lymphoma, or tuberculosis; therefore, histopathological confirmation remains essential in atypical cases (3, 4).

Because no specific serological marker is currently available for sarcoidosis, diagnosis still relies on compatible clinical and radiological findings, histopathological evidence, and exclusion of alternative causes (2). Serum angiotensin-converting enzyme (ACE), serum calcium, urinary calcium, soluble interleukin-2 receptor (sIL-2R), and lysozyme may support the evaluation of suspected sarcoidosis, but none is diagnostic on its own (2, 6, 7). ACE may reflect granuloma burden, although its sensitivity and specificity are variable. Hypercalcemia or hypercalciuria may result from granuloma-driven extrarenal vitamin D activation, but these abnormalities occur only in a subset of patients (2, 7). sIL-2R may reflect T-cell activation and inflammatory or multiorgan disease activity, whereas lysozyme remains non-specific (6, 8). These markers should therefore be interpreted together with clinical, radiological, and pathological findings, and alternative granulomatous diseases must be excluded (2).

Endobronchial ultrasound-guided transbronchial needle aspiration (EBUS-TBNA) has become an important, minimally invasive method for sampling mediastinal and hilar lymph nodes, with good diagnostic performance and safety in suspected sarcoidosis (9). However, diagnostic limitations remain. Granulomatous lesions may be unevenly distributed across nodal stations, and a negative result from a single puncture site does not reliably exclude the disease; accordingly, repeat or multi-station sampling may improve diagnostic yield in clinically suspicious cases (9–11).

Interleukin-6 (IL-6) is a pro-inflammatory cytokine involved in immune activation and may reflect inflammatory activity in sarcoidosis, although it lacks diagnostic specificity (6). Mechanistically, IL-6 can induce hepcidin expression and contribute to inflammation-related iron restriction (12, 13). It may also influence peripheral thyroid hormone metabolism and participate in low T3 syndrome during systemic illness (14, 15). Therefore, IL-6 should be interpreted as an adjunctive inflammatory marker rather than a diagnostic marker for sarcoidosis.

Beyond imaging findings, sarcoidosis may also be accompanied by systemic inflammatory, metabolic, and endocrine disturbances. Anemia is increasingly recognized in chronic inflammatory diseases and may reflect not only absolute iron deficiency but also inflammation-related alterations in iron handling (12, 16). Likewise, low T3 syndrome may occur in severe inflammatory states and has been linked to cytokine-mediated changes in peripheral thyroid hormone metabolism (14). These abnormalities are non-specific in isolation, but when they coexist with atypical thoracic imaging findings, they may provide additional clues to an underlying inflammatory granulomatous disorder. We report a case of sarcoidosis presenting as a unilateral hilar mass with refractory anemia and low T3 syndrome, a combination that initially obscured the diagnosis but ultimately pointed to a broader inflammation-associated clinical phenotype.

## 2 Case presentation

### 2.1 Initial presentation

A 53-year-old woman presented with a 4-day history of fever, chest tightness, and myalgia. She denied cough, sore throat, abdominal pain, diarrhea, nausea, vomiting, chills, arthralgia, or skin rash. She had no history of smoking, occupational exposure, recent travel, or family history of malignancy. Of note, she had a 2-year history of anemia without clinically evident bleeding. Oral iron supplementation had been prescribed during this period, but her hemoglobin showed no meaningful improvement.

An initial chest computed tomography (CT) scan performed at a local hospital showed a left hilar mass, multiple enlarged mediastinal lymph nodes, and small bilateral pleural effusions. Her symptoms did not improve after treatment with piperacillin-tazobactam, and she was transferred to our hospital on 6 October 2024, for further evaluation.

### 2.2 Physical examination

On admission, her temperature was 38.8 °C, pulse rate 98 beats/min, respiratory rate 18 breaths/min, and blood pressure 106/62 mmHg. She was conscious but mildly lethargic. No cyanosis or superficial lymphadenopathy was observed. Chest examination revealed dullness to percussion and decreased breath sounds over the left lower lung field. Cardiac, abdominal, and neurological examinations were unremarkable.

### 2.3 Imaging and laboratory findings

Repeat contrast-enhanced chest CT at our hospital on 6 October confirmed a left hilar mass with multifocal mediastinal lymphadenopathy involving stations 4L, 5, 7, 11L, and other stations (Figures 1A,B). The imaging appearance raised concern for lung cancer with nodal involvement, with possible obstructive pneumonia.

**FIGURE 1:**
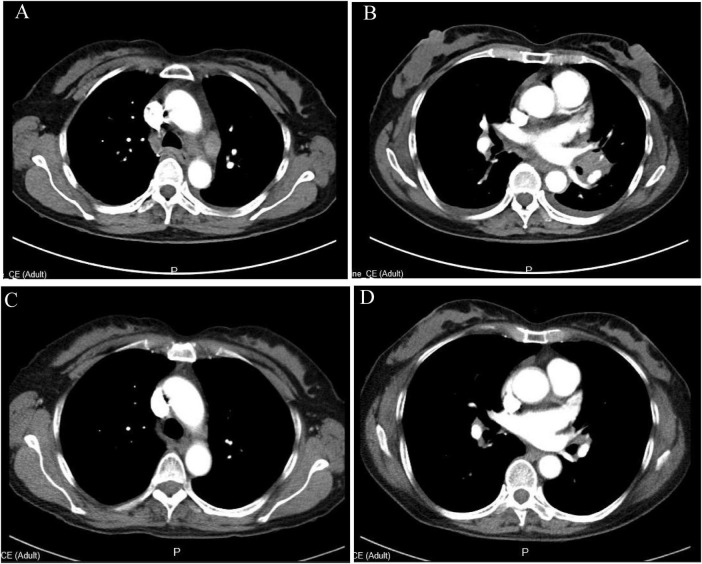
Interval changes in the target mediastinal and left hilar lymph nodes on chest computed tomography (CT). **(A)** Baseline contrast-enhanced chest CT on 6 October 2024, showing enlarged mediastinal lymph nodes at stations 4L and 5. **(B)** Baseline contrast-enhanced chest CT on 6 October 2024, showing an enlarged left hilar lymph node at station 11L. **(C)** Follow-up contrast-enhanced chest CT on 18 November 2024, at a comparable anatomical level, showing marked reduction of the mediastinal lymph nodes at stations 4L and 5. **(D)** Follow-up contrast-enhanced chest CT on 18 November 2024, at a comparable anatomical level, showing shrinkage of the left hilar lymph node at station 11L. Lymphadenopathy in additional stations was identified on sequential imaging slices but is not shown.

Laboratory tests showed pancytopenia, with a white blood cell count of 1.7 × 10^9^/L, hemoglobin of 90 g/L, and platelet count of 70 × 10^9^/L. Inflammatory markers were markedly elevated, including C-reactive protein (CRP) of 98.7 mg/L, interleukin-6 (IL-6) of 60.7 pg/mL, and procalcitonin of 0.5 ng/mL. Thyroid function testing showed low total triiodothyronine (TT3) of 0.57 nmol/L and low free T3 (FT3) of 2.36 pmol/L, with normal total thyroxine (TT4) of 104.2 nmol/L, free T4 (FT4) of 11.87 pmol/L, and thyroid-stimulating hormone (TSH) of 0.90 mIU/L. This pattern was consistent with low T3 syndrome rather than primary thyroid dysfunction. Renal function was preserved, with urea of 3.29 mmol/L and creatinine of 49.2 μmol/L on admission. Serum calcium was slightly reduced at 2.09 mmol/L initially and later normalized to 2.29 mmol/L. Stool routine examination, fecal occult blood testing, and parasite examination were negative, providing no evidence of overt gastrointestinal bleeding, intestinal inflammation, or parasitic infection. Serum tumor markers commonly used in lung cancer evaluation were not elevated. The reference ranges, units, manufacturers, and analytical methods for the laboratory parameters reported in the case presentation are summarized in Supplementary Table 1.

Iron studies showed a discordant pattern: serum iron was reduced to 6.3 μmol/L, whereas ferritin was markedly elevated to 955.2 μg/L. Transferrin was decreased at 1.90 g/L. Together with her long-standing poor response to oral iron therapy, these findings suggested absolute iron deficiency complicated by inflammation-related iron restriction rather than uncomplicated iron deficiency alone.

### 2.4 Diagnostic procedures

Endobronchial ultrasound-guided transbronchial needle aspiration of station seven lymph nodes was performed on 8 October. Cytology showed lymphocytes and bronchial mucosal cells without evidence of malignancy or granulomatous inflammation. This result did not support lymphoma or metastatic carcinoma, but it also failed to establish a definitive diagnosis.

Because the clinical picture remained unresolved, a multidisciplinary reassessment was performed. Ophthalmologic examination showed no evidence of uveitis or retinal involvement. Bone marrow examination demonstrated hyperplastic erythropoiesis with absent iron stores, supporting true iron deficiency, while the high ferritin and low transferrin suggested a superimposed inflammatory component. Endocrinology considered the low T3 level to be compatible with non-thyroidal illness in the setting of systemic inflammation.

Repeat EBUS-TBNA was performed on 11 October, targeting stations 4L and 11L. Histopathological examination revealed epithelioid cell hyperplasia in a lymphocytic background, consistent with non-caseating granulomatous inflammation (Figure 2). Acid-fast staining, fungal staining, and *Mycobacterium tuberculosis* polymerase chain reaction were negative. Additional infectious tests, including T-cell spot test, sputum acid-fast bacilli smear, bronchoalveolar lavage fungal culture, cytomegalovirus testing, and Epstein-Barr virus testing, did not identify an infectious cause. Autoimmune serologies and immunoglobulin levels were unremarkable.

**FIGURE 2:**
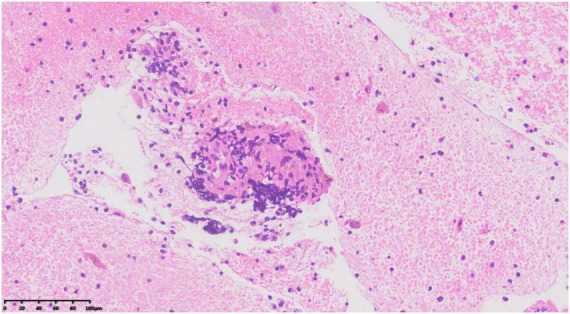
Histopathological findings from endobronchial ultrasound-guided transbronchial needle aspiration (EBUS-TBNA) of stations 4L and 11L. The image demonstrated a non-caseating granuloma with epithelioid cell hyperplasia in a lymphocytic background, consistent with the histopathologic features of sarcoidosis.

### 2.5 Diagnosis and follow-up

Based on the compatible imaging findings, non-caseating granulomatous inflammation, and exclusion of infection and malignancy, the patient was diagnosed with pulmonary sarcoidosis with atypical unilateral hilar and mediastinal lymphadenopathy. Her anemia was considered to reflect mixed absolute iron deficiency and inflammation-related iron dysregulation, and the low T3 syndrome was interpreted as an inflammation-associated endocrine response.

The patient received intravenous iron sucrose and inhaled budesonide-formoterol. On follow-up chest CT on 18 November 2024, the left hilar lesion and mediastinal lymphadenopathy had regressed (Figures 1C,D). Laboratory tests showed normalization of hemoglobin and improvement of inflammatory markers, accompanied by recovery of T3 levels.

## 3 Discussion

This case primarily highlights the diagnostic challenge of unilateral tumor-like hilar sarcoidosis and the value of repeat multi-station EBUS-TBNA when the initial biopsy is non-diagnostic. This presentation is challenging because sarcoidosis typically presents with bilateral hilar and mediastinal lymphadenopathy, whereas unilateral or mass-like thoracic involvement may closely mimic lung cancer, lymphoma, or tuberculosis (3, 17). In this patient, fever, a left hilar mass, mediastinal lymphadenopathy, and pancytopenia created substantial diagnostic uncertainty. This case therefore underscores the need for tissue confirmation when imaging findings are atypical. The key findings supporting the diagnostic and interpretive conclusions are summarized in Supplementary Table 2.

In this case, serological markers were adjunctive rather than diagnostic. Serum calcium was not elevated and normalized during hospitalization, whereas serum ACE, urinary calcium, sIL-2R, and lysozyme were not available, which should be acknowledged as a limitation. Even if abnormal, these markers would not have been sufficient to establish sarcoidosis in isolation, particularly in a unilateral tumor-like hilar presentation that closely mimicked malignancy or infection. Histopathological confirmation and exclusion of infection, malignancy, and other granulomatous diseases therefore remained essential.

Unilateral hilar lymphadenopathy is an uncommon but well-documented atypical presentation of sarcoidosis, reported in approximately 3%–5% of cases in Western cohorts (18). When present, it may mimic malignancy or infection, particularly lung cancer, lymphoma, or tuberculosis (4). In typical cases, symmetric bilateral hilar and mediastinal lymphadenopathy may favor sarcoidosis; however, this clue may be absent in unilateral presentations. Other CT features that may raise suspicion for sarcoidosis include smooth nodal margins, preservation of adjacent bronchovascular structures, and absence of nodal necrosis or cystic change on contrast-enhanced CT (4, 5, 19). Importantly, fluorodeoxyglucose positron emission tomography/computed tomography may show intense nodal uptake in sarcoidosis and therefore overlaps with malignancy, making histopathological confirmation essential when imaging findings are inconclusive (20).

The main diagnostic lesson of this case is that a single negative EBUS-TBNA result should be interpreted cautiously when clinical suspicion persists. Initial sampling from station seven was non-diagnostic, whereas repeat sampling from stations 4L to 11L revealed non-caseating granulomatous inflammation and established the diagnosis. This suggests that granulomatous involvement may be unevenly distributed across lymph node stations, particularly in atypical unilateral or mass-like disease. For clinicians evaluating unexplained mediastinal or hilar lymphadenopathy, repeat multi-station EBUS-TBNA may reduce false-negative interpretation and prevent premature exclusion of sarcoidosis.

The patient’s iron profile provided an additional clue to systemic inflammation but should not be viewed as a novel paradoxical phenomenon. She had low serum iron, markedly elevated ferritin, reduced transferrin, elevated CRP, and increased IL-6. This pattern is not typical of uncomplicated iron deficiency anemia, in which ferritin is usually low. However, bone marrow examination showed absent iron stores, confirming true iron deficiency. Thus, the anemia was best interpreted as mixed absolute iron deficiency and inflammation-related iron restriction. Mechanistically, IL-6-mediated hepcidin activation may plausibly explain the inflammation-related iron restriction observed in this patient. Previous studies have shown that IL-6 can induce hepatic hepcidin expression through the Janus kinase/signal transducer and activator of transcription three pathway, and hepcidin can limit circulating iron availability by reducing intestinal iron absorption and iron release from macrophages (13, 21). However, because serum hepcidin was not measured, this pathway remains hypothetical in the present case and should not be interpreted as directly demonstrated.

The low T3 syndrome may also have reflected systemic inflammation. Non-thyroidal illness syndrome is well-recognized in acute and chronic systemic illness (22, 23), and IL-6 has been shown to interfere with peripheral thyroid hormone metabolism (15). In this patient, the thyroid panel showed low TT3 and FT3 with preserved TT4, FT4, and TSH, favoring non-thyroidal illness syndrome rather than primary thyroid disease. Renal function was preserved, and stool routine examination, fecal occult blood testing, and parasite examination were negative. Although formal malabsorption testing was not performed, the available findings did not suggest gastrointestinal blood loss, intestinal inflammation, parasitic infection, or renal dysfunction as major alternative explanations. The low T3 level improved alongside the inflammatory state, supporting a reversible inflammation-associated process.

In summary, this patient presented with sarcoidosis in an unusual form: a unilateral tumor-like hilar lesion, mixed iron deficiency and inflammation-related iron restriction, and low T3 syndrome. The case reminds clinicians that low serum iron with elevated ferritin, especially after poor response to oral iron therapy, should prompt assessment for both absolute iron deficiency and inflammation-related iron restriction. When the first EBUS-TBNA is non-diagnostic but clinical suspicion remains, repeat sampling from additional nodal stations may be considered in selected cases.

## Data Availability

The raw data supporting the conclusions of this article will be made available by the authors, without undue reservation.
