# Supplementary material for: Unilateral hilar sarcoidosis with anemia and low T3 syndrome: a case report
Source: Front Med (Lausanne). 2026 Jul 8;13:1892525. doi: 10.3389/fmed.2026.1892525 (PMC13388458; doi:10.3389/fmed.2026.1892525)
Supplement: Supplementary file 1 [file Table_1.docx]

| Supplementary Table 1. Reference ranges, units, manufacturers, and analytical methods of laboratory parameters reported in the case presentation. | | | | |
| --- | --- | --- | --- | --- |
| Parameter | Reference range | Unit | Manufacturer/brand | Method |
| White blood cell count | 4.0–10.0 | ×10^9/L | Mindray | Flow fluorescence scattering |
| Hemoglobin | 113–151 | g/L | Mindray | Colorimetric method |
| Platelet count | 100–300 | ×10^9/L | Mindray | Hydrodynamic focusing method |
| C-reactive protein | <10.0 | mg/L | Mindray | Immunoturbidimetric assay |
| Interleukin-6 | <7 | pg/mL | Roche | Electrochemiluminescence immunoassay |
| Total triiodothyronine | 0.98–2.33 | nmol/L | Abbott | Chemiluminescent immunoassay |
| Free triiodothyronine | 2.43–6.01 | pmol/L | Abbott | Chemiluminescent immunoassay |
| Total thyroxine | 62.7–150.8 | nmol/L | Abbott | Chemiluminescent immunoassay |
| Free thyroxine | 9.01–19.05 | pmol/L | Abbott | Chemiluminescent immunoassay |
| Thyroid-stimulating hormone | 0.35–4.94 | mIU/L | Abbott | Chemiluminescent immunoassay |
| Urea | 2.80–7.60 | mmol/L | Johnson & Johnson | Dry chemistry method |
| Creatinine | 41.0–72.0 | μmol/L | Johnson & Johnson | Dry chemistry method |
| Serum calcium | 2.11–2.52 | mmol/L | Johnson & Johnson | Dry chemistry method |
| Fecal occult blood test | Negative | — | Keyu | Automated stool analyzer |
| Parasite examination | None detected | — | Not specified | Microscopic examination |
| Carcinoembryonic antigen | <5 | ng/mL | Abbott | Chemiluminescent immunoassay |
| Cytokeratin 19 fragment | <5 | ng/mL | Tellgen | Chemiluminescent immunoassay |
| Neuron-specific enolase | <25 | ng/mL | Tellgen | Chemiluminescent immunoassay |
| Squamous cell carcinoma antigen | <1.5 | ng/mL | Abbott | Chemiluminescent immunoassay |
| Serum iron | 7.0–30.0 | μmol/L | Leadman | Ferrozine method |
| Ferritin | 10.0–291.0 | μg/L | Siemens | Chemiluminescent immunoassay |
| Transferrin | 2.00–3.60 | g/L | Siemens | Immunoturbidimetric assay |

| Supplementary Table 2. Key findings and their diagnostic or interpretive implications | |
| --- | --- |
| Key findings | Diagnostic or interpretive implication |
| Unilateral left hilar mass and mediastinal lymphadenopathy | Atypical thoracic presentation requiring exclusion of malignancy, lymphoma, and tuberculosis |
| Initial station 7 EBUS-TBNA was nondiagnostic, followed by non-caseating granulomatous inflammation on repeat sampling of stations 4L and 11L | Supported sarcoidosis after repeat multi-station sampling |
| Negative microbiological tests, including acid-fast staining, fungal staining, tuberculosis PCR, and fungal culture | Infection, especially tuberculosis and fungal disease, was not supported |
| Tumor markers were not elevated, and cytology showed no malignant cells | Malignancy was not supported by the available evaluation |
| Low serum iron, markedly elevated ferritin, reduced transferrin, elevated CRP/IL-6, and absent bone marrow iron stores | Suggested absolute iron deficiency with inflammation-related iron restriction |
| Low total T3 with recovery during follow-up | Compatible with reversible non-thyroidal illness in systemic inflammation |
| Regression of hilar and mediastinal lesions on follow-up CT | Provided supportive follow-up evidence for sarcoidosis |

Mechanistic interpretations regarding inflammation-related iron restriction and low T3 syndrome were based on the combination of findings in this case and established literature; they were not considered direct mechanistic proof.
